# Antinociceptive and anti-inflammatory effects of* Urtica dioica* leaf extract in animal models

**Published:** 2013

**Authors:** Valiollah Hajhashemi, Vahid Klooshani

**Affiliations:** 1 ***Department of Pharmacology and Isfahan Pharmaceutical Sciences Research Center, School of Pharmacy and Pharmaceutical Sciences, Isfahan University of Medical Sciences, Isfahan, I. R. Iran***

**Keywords:** Anti-inflammatory, Antinociceptive, Extract, *Urtica dioica*

## Abstract

**Objective:** This study was aimed to examine the antinociceptive and anti-inflammatory effects of *Urtica dioica* leaf extract in animal models.

**Materials and Methods:** Hydroalcoholic extract of the plant leaves was prepared by percolation method. Male Swiss mice (25-35 g) and male Wistar rats (180-200 g) were randomly distributed in control, standard drug, and three experimental groups (n=6 in each group). Acetic acid-induced writhing, formalin test, and carrageenan-induced paw edema were used to assess the antinociceptive and anti-inflammatory effects.

**Results:** The extract dose-dependently reduced acetic acid-induced abdominal twitches. In formalin test, the extract at any of applied doses (100, 200, and 400 mg/kg) could not suppress the licking behavior of first phase while doses of 200 and 400 mg/kg significantly inhibited the second phase of formalin test. In carrageenan test, the extract at a dose of 400 mg/kg significantly inhibited the paw edema by 26%.

**Conclusion:** The results confirm the folkloric use of the plant extract in painful and inflammatory conditions. Further studies are needed to characterize the active constituents and the mechanism of action of the plant extract.

## Introduction

*Urtica dioica *L. (*U. dioica*) or stinging nettle is a member of the Urticaceae family. This herbaceous perennial flowering plant grows in many regions of the world. In Iran, it grows widely in Northern provinces. The plant has many stinging hairs on its leaves and stem and when touched injects into the skin several chemicals including acetylcholine, histamine, 5-HT (serotonin), moroidin, leukotrienes, and possibly formic acid and causes a painful sting or paresthesia from which the species derives its common name "stinging nettle" (Bisser, 1994 ▶; Newall et al., 1996 ▶; Iranian Herbal Pharmacopoea Committee, 2003 ▶). In Persian, the common name of the plant is "gazaneh" which also means stinging. *U. dioica* leaves contain chlorophyll, carotene, xanthophil, and flavonoid compounds (Iranian Herbal Pharmacopoea Committee, 2003 ▶). The root contains tannins, coumarin (scopoletin), triterpens, lignans, lectins, sterols (sitosterol, campesterol, and stigmasterol), and flavonoids (Bisser, 1994 ▶). Polysaccharides and caffeic malic acid are both found to some extent in all parts of *U. dioica* and demonstrated anti-inflammatory activity in an *in vitro* study (Obertreis et al., 1996 ▶).

Traditionally the plant has been used as diuretic, anti-inflammatory, and aphrodisiac as well as a treatment for gout, hair loss, and mild bleeding (Grieve M, 1971 ▶; Iranian Herbal Pharmacopoea Committee, 2003 ▶). 

Antibacterial as well as antifungal effects have been reported for *U. dioica* extracts (Iranian Herbal Pharmacopoea Committee, 2003 ▶; Dar et al., 2012) and it is used in shampoos to control dandruff (). Several double-blind clinical trials confirmed the efficacy of *U. dioica* root for relieving the symptoms of benign prostatic hyperplasia (BPH) (Safarinejad, 2005 ▶). It has been reported that nettle root contains lignan compounds such as 3,4-divanillyltetrahydrofuran which modulate binding of sex hormone binding globulin (SHBG) to its receptors on prostate cell membranes (Hryb et al., 1995 ▶; Schottner et al., 1997 ▶). It has also been shown that the steroidal compounds stigma sterol, stimast-4-en-3-one, and campesterol can inhibit the prostatic sodium/potassium pump, which might contribute to nettle’s effects in BPH (Hirano et al., 1994 ▶). In animal studies, *U. dioica* extract showed inhibition of platelet aggregation and improvement of lipid profiles such as decrease of total and LDL cholesterol, plasma Apoprotein B, and the LDL/HDL ratio (Daher et al., 2006 ▶; El Haouari., 2006 ▶).

Based on above information and also a report indicating antinociceptive and anti-inflammatory activities of another species of Urtica (*Urtica urens*) (Marrassini et al., 2010), this work was designed to study the antinociceptive and anti-inflammatory effects of orally administered *U. dioica* in animal models and to find pharmacological evidence for its folkloric use in painful and inflammatory disorders.

## Materials and Methods

Plant material and preparation of extract 

*U. dioica* L. leaves were collected from Sari, Iran in 2011. The plant was confirmed by the herbarium department of Barij Essence Company (Kashan, Iran) and a reference specimen (No. 1-204) of the plant was deposited there. For preparation of hydroalcoholic extract, powdered leaves (500 g) were macerated with ethanol: water (1:1) for 2 days. The extract was then shaked, filtered, and dried in a freeze-dryer (Sajjadi et al., 1998 ▶). The yield was 12%. 

**Chemicals**

Lambda carrageenan and indomethacin were purchased from Sigma Chemical Company (St. Louis, USA). Acetic acid and formalin (Merck, Germany) were used in pain models. Morphine sulfate was purchased from Tolid Daru, Iran. 

**Animals**

Analgesic tests were carried out on male Swiss mice (25-35 g). Male Wistar rats (180-200 g) were used for carrageenan test. In all experiments, each group consisted of 6 animals. Animals were housed in standard cages, on 12 h light/dark cycle and air temperature was maintained at 22 ± 2 ^°^C with free access to food and water *ad libitum. *They were acclimatized to laboratory conditions for at least one week before testing. All experiments were performed according to guidelines for the care of laboratory animals of Ethics Committee of Isfahan University of Medical Sciences.

**Acetic acid-induced writhing test**

This test was carried out according to Koster et al. method (Koster et al., 1959). Groups of mice (n=6) received different doses of *U. dioica* leaf extract (100-400 mg/kg, p.o., by means of a stomach tube) 45 min prior to an intraperitoneal injection of acetic acid 1% in a volume of 10 ml/kg. Control group received vehicle (10 ml/kg saline). Indomethacin (10 mg/kg, i.p.) was used as the reference drug. Number of abdominal twitches (writhes) counted in each 10 min period starting 10 min after acetic acid injection.

**Formalin test**

This test was carried out according to Hunskaar and Hole (1987) method. Groups of mice (n=6) were orally administered different doses (100, 200, and 400 mg/kg) of hydroalcoholic extract of *U. dioica* leaves 45 min prior to injection of 20 l of 2.5% formalin (v/v in 0.9% saline) into the subplantar space of the right hind paw. Control group received vehicle (10 ml/kg of saline). Morphine (10 mg/kg, i.p.) was used as the standard analgesic drug. The duration of paw licking was determined 0-5 min (first phase) and 20-30 min (second phase) after formalin for each mouse. 

**Carrageenan-induced rat paw edema**

The anti-inflammatory activity was evaluated by the carrageenan-induced paw edema test in the rats (Vogel and Vogel, 1997 ▶). After a light anaesthesia of rats with diethylether, 0.1 ml of a freshly prepared suspension of lambda carrageenan (1% w/v) in isotonic saline was injected into the subplantar space of the right hind paw of rats. The left hind paws were injected with the same volume of saline and used as the control. Paw volume was measured prior and 4 h after carrageenan administration using a mercury plethysmorgraph (Ugo Basil, Italy). 

Hydroalcoholic extract of the plant (200 and 400 mg/kg) was orally administered 45 min prior to carrageenan administration. The control group received equal volume of the vehicle. Indomethacin (10 mg/kg, p.o.) was used as positive control.

**Statistical analysis**

Data were analyzed by SPSS (version 13) using one way analysis of variance (ANOVA) followed by Scheffe post hoc test. The results were expressed as mean±SEM and p-values less than 0.05 were considered significant.

## Results

**Figure 1:**
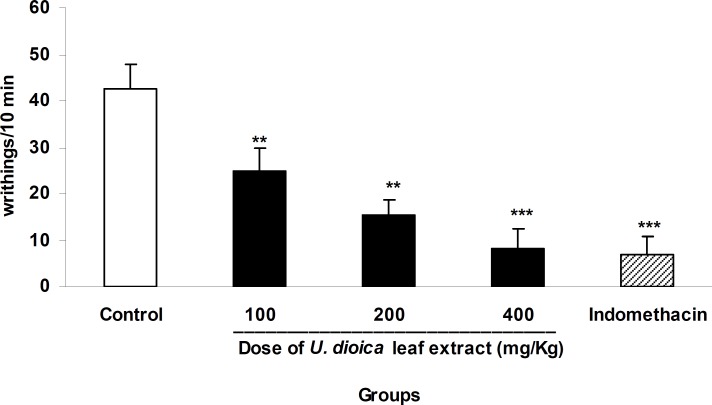
The antinociceptive activity of *U. dioica* leaf extract in acetic acid-induced writhing test. Vehicle and different doses of the extract (100, 200, and 400 mg/kg) were orally administered 45 min prior to i.p. injection of acetic acid 1% (10 ml/kg) and the number of abdominal twitches was counted in a 10 min period starting 10 min after acetic acid injection. Data are mean±SEM of 6 animals in each group. ** p<0.01 and *** p<0.001 significantly different from control group

In acetic acid-induced writhing test, *U. dioica* leaf extract at doses of 100, 200, and 400 mg/kg inhibited abdominal twitches by 41%, 64% and 81%, respectively. Indomethacin as the reference analgesic reduced the twitches by 84% (Figure 1, p<0.001).

**Figure 2:**
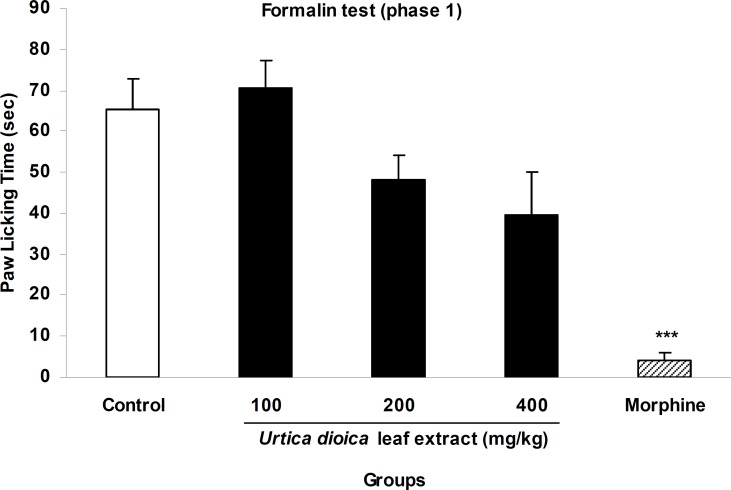
The antinociceptive activity of *U. dioica* leaf extract on paw licking during acute phase of formalin test. Vehicle and different doses of the extract (100, 200, and 400 mg/kg) were orally administered 45 min prior to subplantar injection of formalin and the time spent for licking was measured during a 0-5 min period starting after formalin injection. Morphine (10 mg/kg, i.p.) was used as reference drug. Data are mean±SEM of 6 animals in each group. *** p<0.001 significantly different from control group

**Figure 3:**
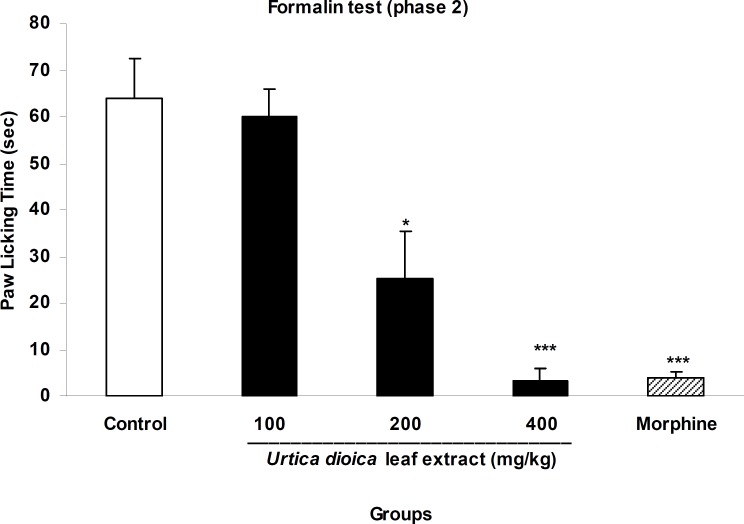
The antinociceptive activity of *U. dioica* leaf extract on paw licking during chronic phase of formalin test. Vehicle and different doses of the extract (100, 200, and 400 mg/kg) were orally administered 45 min prior to subplantar injection of formalin and the time spent for licking was measured during a 20-30 min period starting after formalin injection. Morphine (10 mg/kg, i.p.) was used as reference drug. Data are mean±SEM of 6 animals in each group. * p<0.05 ;*** p<0.001 significantly different from control group

In acute phase of formalin test, the extract at a dose of 100 mg/kg did not show any analgesia while doses of 200 and 400 mg/kg produced 26% and 39.8% inhibition of licking behavior (Figure 2). In chronic phase, above doses of the extract inhibited paw licking by 6%, 60.2%, and 94.8%, respectively and these changes were significant at doses of 200 and 400 mg/kg compare with the control group (p<0.05 and p<0.001, respectively). Morphine (10 mg/kg, i.p.) as the standard analgesic drug inhibited both acute and choronic phases of formalin test by 93.6% and 93.7%, respectively (Figures 2 and 3).

**Figure 4:**
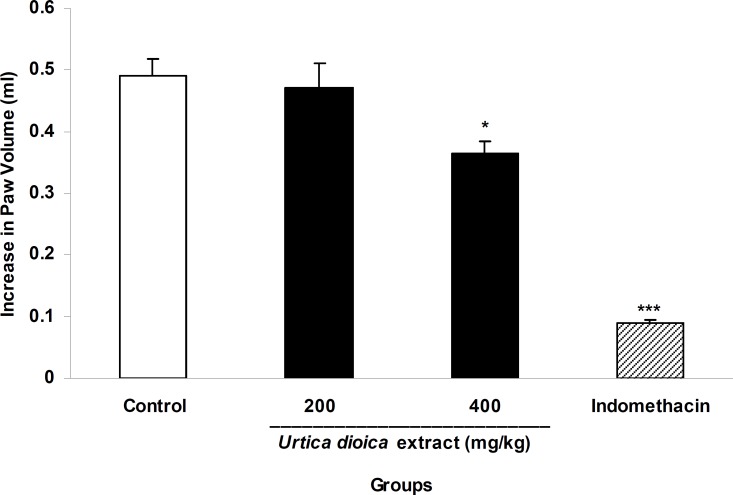
The anti-inflammatory activity of *U. dioica* leaf extract in carrageenan-induced paw edema. Vehicle, reference drug (Indomethacin,10 mg/kg) and two doses of the extract (200 and 400 mg/kg) were orally administered 45 min prior to subplantar injection of carrageenan (0.1 ml, 1% w/v) and the paw volume was measured before and 4 h after carrageenan. Data are mean ± SEM of 6 animals in each group. * p<0.05 and *** p<0.001 significantly different from control group

In carrageenan-induced paw edema, the extract at a dose of 400 mg/kg significantly (p<0.05) reduced inflammation. Indomethacin (10 mg/kg, i.p.) also exerted a significant (p<0.001) inhibition of carrageenan-induced edema (Figure 4).

## Discussion

In the present study, *U. dioica *leaf extract showed analgesic activity in acetic acid-induced writhing and formalin tests. Acetic acid-induced abdominal pain has similarity with human visceral disorders and it has been extensively used for screening of analgesic drugs (Vogel & Vogel, 1997 ▶). In this test, many drugs including opioids, non-steroidal anti-inflammatory drugs (NSAIDs), antispasmodics, calcium channel blockers, and antihistamines show analgesic activity (Collier et al., 1968 ▶; Vogel & Vogel, 1997 ▶).

In the formalin pain model, formalin induced the typical biphasic pain response (Hunskaar and Hole, 1987 ▶). High nociceptive scores were recorded during the first 5 min (first phase or acute phase) after subcutaneous formalin administration and were followed by a reduction in scores for several minutes. The second phase (chronic phase) was also started 20 min after formalin injection. Pain in the acute phase is mainly caused by activation of C-fibers, while in the late phase a combination of an inflammatory reaction in the peripheral tissue and functional changes in the dorsal horn of the spinal cord are involved (Tjolsen et al., 1992 ▶). 

In this study, hydroalcoholic extract of *U. dioica *leaves reduced the pain response of the second phase of formalin test. The late (second) phase is inflammatory in origin (Chen et al., 1995 ▶; Elisabetsky et al., 1995 ▶) and the extract showed considerable analgesia in this phase and therefore it may indicate an anti-inflammatory effect of the leaves of the plant. These results are in agreement with those which were reported for *U. urens* (another species of Urtica) (Marrassini et al., 2010). Results of carrageenan-induced paw edema which is a routine and valid animal model for assessing anti-inflammatory activity also confirm the anti-inflammatory effect *U. dioica *leaf extract. Although the extract at doses of 200 and 400 mg/kg inhibited the licking behavior of animals by about 40%, but this effect was not statistically significant. It has been reported that centrally acting analgesic drugs such as opioids are able to inhibit the pain response of the first phase of formalin test (Chen et al., 1995 ▶; Elisabetsky et al., 1995 ▶).

We have previously reported anti-inflammatory and antinociceptive activities for flavonoids and polyphenolic compounds of other plants (Ghannadi et al., 2005 ▶; Hajhashemi et al., 2003 ▶, 2009, 2011) and it seems that the pharmacological effects observed in the present study may be partially due to flavonoids and polyphenoilc contents of *U. dioica* extract. The antinociceptive and anti-inflammatory effects of flavonoids and polyphenolic compounds may be attributed to their antioxidant activity (Bors and Saran, 1987 ▶), inhibition of histamine release from mast cells and inhibition of arachidonic acid metabolism (Amresh et al., 2007 ▶). In general, inflammation is a complex process which results from involvement of many mediators and further studies are required to find out the exact mechanism of *U. dioica* leaf extract. 

Taking into account the above results, it can be concluded that *U. dioica *leaf extract has considerable anti-inﬂammatory and analgesic activities and this study provides pharmacological evidence for its folkloric use in arthritis and other inflammatory complications. 
